# Trends in incidence and demographics of testicular cancer in California, 2000–2020

**DOI:** 10.1002/bco2.451

**Published:** 2024-10-30

**Authors:** David J. Benjamin, Anshu Shrestha, Dimitra Fellman, Arash Rezazadeh Kalebasty

**Affiliations:** ^1^ Hoag Family Cancer Institute Newport Beach California USA; ^2^ Cancer Registry of Greater California (CRGC) Public Health Institute Sacramento California USA; ^3^ Department of Medicine, Division of Hematology and Oncology, Chao Family Comprehensive Cancer Center University of California, Irvine Orange California USA

**Keywords:** California, incidence, race/ethnicity, testicular cancer, trends

## 1 INTRODUCTION

The incidence of testicular cancer has been rising globally among young adult men for the past five decades for reasons not currently well‐understood.^1^ Although a rare genitourinary malignancy that is generally curable, testicular cancer remains a significant public health concern due to long‐term medical, psychological and social burden associated with treatment and its short‐ and long‐term toxicities.^2^ Risk factors leading to the development of testicular cancer include age, family or personal history of testicular cancer, cryptorchidism, race/ethnicity and recreational drug use such as marijuana.^1^

White males have historically had the highest incidence rates of testicular cancer, while Black males have the lowest incidence rates. However, data from 2001 to 2016 extracted from the National Cancer Institute's Surveillance, Epidemiology, and End Results (SEER) programme demonstrated that the incidence of testicular cancer was rising throughout the United States and Asian/Pacific Islander men had the largest increases in incidence followed by Hispanic men.^3^ Given that California is the most populous state in the United States and one of the most racially/ethnically diverse populations, we sought to evaluate trends in demographics including race/ethnicity from updated population data up to the year 2020.

## 2 METHODS

Males diagnosed with testicular cancer between 2000 and 2020 were identified through the California Cancer Registry (CCR) database, one of the largest cancer registries in the United States. Cases were excluded if the age at the time of diagnosis was unknown. Incidence rates per 100 000, stratified by year of diagnosis, race/ethnicity and age were calculated, and age‐adjusted to the 2000 US Standard Population. This study involved analysis of de‐identified data from the state‐mandated cancer registry database and as such, does not require patient informed consent. Therefore, the study was exempt from Institutional Review Board (IRB) approval.

## 3 RESULTS

We identified a total of 23 214 cases of testicular cancer during the study period. Most men (71.5%, *n* = 16 599) were below age 40 at the time of diagnosis. The majority of men were non‐Hispanic white (52.5%, *n* = 12 191), followed by Hispanic (37.6%, *n* = 8720), Asian/Pacific Islander (5.0%, *n* = 1170) and non‐Hispanic Black (1.8%, *n* = 422). Testicular cancer diagnoses were equally distributed between neighbourhood socio‐economic status (nSES) groups (highest quintile (20.3%), upper‐middle (21.3%), middle (21.0%), lower‐middle (20.2%) and lowest (17.3%)). Additional demographic information including marital status and Charlson comorbidity index are available in Table S1.

Testicular cancer incidence rate rose among all racial/ethnic groups in California between 2000 and 2020. The rates rose at a faster pace among Hispanic and Asian/Pacific Islander men during this period. Among Hispanic men, the incidence rate was 4.2/100 000 in 2000 and rose to 6.7/100 000 in 2020. Similarly, the incidence rate for Asian/Pacific Islander men was 2.0/100 000 in 2000, decreased to 1.2/100 000 in 2002 and rose to 2.5/100 000 in 2020. In contrast, the incidence rate was the highest among non‐Hispanic white men at 7.3/100 000 in 2000 and remained at a similar rate at 7.6/100 000 in 2020. Similarly, among non‐Hispanic Black men, the incidence rate remained stable with a rate of 1.7/100 000 in 2000 compared to a rate of 2.0/100 000 in the year 2020. Trends in incidence between 2000 and 2020 by race/ethnicity are shown in Figure S1.

**FIGURE 1:**
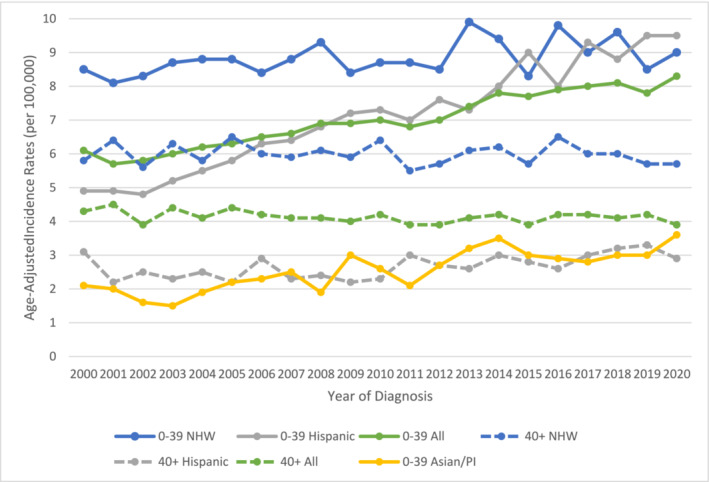
Incidence rate trends for male malignant testicular cancer by age and race/ethnicity in California, 2000–2020.

When assessing by age, the increasing trend is mainly observed in the younger population (<40) as opposed to 40 years or older men. For example, the incidence rate among men under 40 at the time of diagnosis rose from 6.1/100 000 in 2000 to 8.3/100 000 in 2020. In contrast, the incidence rate of testicular cancer in men 40 or older was 5.7/100 000 in 2000 and down to 4.9/100 000 in 2020. Increasing trend in men under 40 are more pronounced in Hispanic and Asian/Pacific Islander compared to non‐Hispanic whites (see Figure 1). Incidence rates in Hispanic men under 40 increased from 4.9/100 000 in 2000 to 9.5/100 000 in 2020, and from 2.1/100 000 in 2000 to 3.6/100 000 in 2020 for Asians/Pacific Islanders. Incidence rates for non‐Hispanic whites remained relatively unchanged during the same time period, fluctuating between 8.1/100 000 and 9.3/100 000.

## 4 DISCUSSION

The incidence of testicular cancer has been rising in California since 2000, particularly among Hispanic and Asian/Pacific Islander men as well as among men who are below the age of 40, consistent with previous studies in the United States. To our knowledge, two other studies have demonstrated a similar rise in incidence among Hispanic and Asian/Pacific Islander men in the United States between the years 1973–2012 and 2001–2016, respectively.^3^, ^4^ This study confirms this trend among Hispanic and Asian/Pacific Islander men with additional follow‐up to the year 2020.

The underlying causes of such rising trends in testicular cancer remain unknown, although some have suggested environment might be a potential aetiology. Cancer registry data from *Cancer Incidence in Five Continents* demonstrated that the highest incidence of testicular cancer between 1978 and 2021 remains in Europe, with Croatia experiencing the highest average annual percentage change during this time period.^5^ Latin American and Caribbean countries experienced annual percentage changes ranging from 2.1% to 4.1%, while several Asian countries such as India, Philippines, and Thailand experienced decreases in annual incidence during the study time period. The discordance in findings of incidence among Hispanic and Asian/Pacific Islander men in native countries compared to California reinforces previous concerns over lifestyle and environmental factors as a possible explanation for the rise in testicular cancer incidence in certain regions.^1^, ^6^

In fact, a prior study conducted in Denmark evaluated the risk of testicular cancer among first‐generation and subsequent generation immigrants compared to native‐born Danes, and found that while the risk of testicular cancer risk was lower in first‐generation immigration compared to native Danes, the risk in second‐generation immigrants was similar to native Danes.^7^ The study's findings suggest that environmental factors likely influence the risk of developing testicular cancer. In fact, population‐based studies have suggested that maternal factors including the in utero environment, excess endogenous hormones such as oestrogen during pregnancy, maternal age, and maternal smoking may lead to an increased risk for development of testicular cancer in male offspring; however, inconsistencies between studies have hindered the ability to come to definitive conclusions.^8^

We used population‐based registry data from one of the most populous and diverse states. This allowed us to examine incidence rates even in small subgroups such as Asian/Pacific Islander and <40 age group. However, one limitation of this study was the inability to evaluate individual‐level potential risk factors including family or personal history of testicular cancer, or cryptorchidism, and how they may be associated with the increases in testicular incidence, particularly among Asian/Pacific Islander and Hispanic men. Moreover, due to a lack of immigration status in the data, it was not possible to evaluate whether first‐generation immigrant status versus second‐ or later‐generation immigrant status could explain the rises in incidence rates among Asian/Pacific Islander and Hispanic men.

## 5 CONCLUSION

The incidence of testicular cancer is rising in California between 2000 and 2020. While white men continue to have the highest incidence rate of testicular cancer, Asian/Pacific Islander and Hispanic men are experiencing increases in incidence rates. Further research particularly into environmental and maternal factors may provide additional information on the aetiology of this growing public health concern.

## AUTHOR CONTRIBUTIONS

**David J. Benjamin:** Conceptualization; formal analysis; writing—original draft; writing—review and editing. **Anshu Shrestha:** Data collection; formal analysis; writing—original draft; writing—review and editing. **Dimitra Fellman:** Data collection; formal analysis; writing—original draft; writing—review and editing. **Arash Rezazadeh Kalebasty:** Conceptualization; formal analysis; writing—original draft; writing—review and editing; supervision.

## DISCLOSURE OF INTEREST

D.J.B. has the following disclosures: Consulting or Advisory Role: AIMED BIO, Astellas, Eisai, Seagen. Speakers' Bureau: Merck. Travel and Accommodations: Merck, Seagen. A.R.K. has the following disclosures: Stock and Other Ownership Interests: ECOM Medical. Consulting or Advisory Role: Exelixis, AstraZeneca, Bayer, Pfizer, Novartis, Genentech, Bristol Myers Squibb, EMD Serono, Immunomedics, Gilead Sciences. Speakers' Bureau: Janssen, Astellas Medivation, Pfizer, Novartis, Sanofi, Genentech/Roche, Eisai, AstraZeneca, Bristol Myers Squibb, Amgen, Exelixis, EMD Serono, Merck, Seattle Genetics/Astellas, Myovant Sciences, Gilead Sciences, AVEO. Research Funding: Genentech, Exelixis, Janssen, AstraZeneca, Bayer, Bristol Myers Squibb, Eisai, Macrogenics, Astellas Pharma, BeyondSpring Pharmaceuticals, BioClin Therapeutics, Clovis Oncology, Bavarian Nordic, Seattle Genetics, Immunomedics, Epizyme. Travel, Accommodations, Expenses: Genentech, Prometheus, Astellas Medivation, Janssen, Eisai, Bayer, Pfizer, Novartis, Exelixis, AstraZeneca. The remaining authors (A.S. and D.F.) have no disclosures.

## Supporting information


**Table S1.** Descriptive Data on Male Malignant Testicular Cancer in California, 2000–2020.

**Figure S1.** Age‐Adjusted Incidence Rates of Testicular Cancer by Race/Ethnicity in California, 2000–2020.
