# Supplementary material for: Trends in incidence and demographics of testicular cancer in California, 2000–2020
Source: BJUI Compass. 2024 Oct 30;5(12):1249–51. doi: 10.1002/bco2.451 (PMC11685165; doi:10.1002/bco2.451)
Supplement: Supplementary file 1 — Table S1. Descriptive Data on Male Malignant Testicular Cancer in California, 2000–2020. [file BCO2-5-1249-s001.docx]

**Table S1. Descriptive Data on Male Malignant Testicular Cancer in California, 2000-2020**

| **Category** | **Value** | **Count** | **Percentage** |
| --- | --- | --- | --- |
| **Age Category** |  |  |  |
|  | 0-39 | 16,599 | 71.5% |
|  | 40+ | 6,615 | 28.5% |
| **Race/Ethnicity** | Non-Hispanic White | 12,191 | 52.5% |
|  | Non-Hispanic Black | 422 | 1.8% |
|  | Hispanic | 8,720 | 37.6% |
|  | Asian/Pacific Islander | 1,170 | 5.0% |
|  | Other/Unknown | 711 | 3.1% |
| **Marital Status** | Single/Separated/Divorced/Widowed | 12,855 | 55.4% |
|  | Married/Domestic Partner | 9,132 | 39.3% |
|  | Unknown | 1,227 | 5.3% |
| **Charlson Comorbidity Index** | 0 | 12,834 | 55.3% |
|  | 1-2 | 1,848 | 8.0% |
|  | 3+ | 213 | 1.0% |
|  | Unknown | 8,319 | 35.8% |
| **Neighborhood SES** | Lowest | 4,012 | 17.3% |
|  | Lower-Middle | 4,683 | 20.1% |
|  | Middle | 4,881 | 21.0% |
|  | Upper-Middle | 4,932 | 21.3% |
|  | Highest | 4,706 | 20.3% |
