# Supplementary material for: Trends in incidence and demographics of testicular cancer in California, 2000–2020
Source: BJUI Compass. 2024 Oct 30;5(12):1249–51. doi: 10.1002/bco2.451 (PMC11685165; doi:10.1002/bco2.451)
Supplement: Supplementary file 2 — Figure S1. Age‐Adjusted Incidence Rates of Testicular Cancer by Race/Ethnicity in California, 2000–2020. [file BCO2-5-1249-s002.docx]

**Figure S1. Age-Adjusted Incidence Rates of Testicular Cancer by Race/Ethnicity in California, 2000-2020**

Legend: NHW – Non-Hispanic White; NHB – Non-Hispanic Black; PI – Pacific Islander
